# Systematic protein-protein interaction and pathway analyses in the idiopathic inflammatory myopathies

**DOI:** 10.1186/s13075-016-1061-7

**Published:** 2016-07-07

**Authors:** Joanna E. Parkes, Simon Rothwell, Philip J. Day, Neil J. McHugh, Zoë E. Betteridge, Robert G. Cooper, William E. Ollier, Hector Chinoy, Janine A. Lamb, The Myositis Genetics Consortium (MYOGEN)

**Affiliations:** Centre for Epidemiology, University of Manchester, 2.722 Stopford Building, Oxford Road, Manchester, M13 9PT UK; Centre for Genetics and Genomics, Arthritis Research UK, University of Manchester, Manchester, UK; Manchester Institute of Biotechnology, University of Manchester, Manchester, UK; Bath Institute of Rheumatic Diseases, Royal National Hospital for Rheumatic Diseases, Bath, UK; MRC/ARUK Institute of Ageing and Chronic Disease, University of Liverpool, Liverpool, UK; National Institute of Health Research Manchester Musculoskeletal Biomedical Research Unit, Central Manchester University Hospitals NHS Foundation Trust, University of Manchester, Manchester, UK

**Keywords:** Idiopathic inflammatory myopathies, Autoantibodies, Association, Protein-protein interaction, Pathway analysis

## Abstract

**Background:**

The idiopathic inflammatory myopathies (IIM) are autoimmune diseases characterised by acquired proximal muscle weakness, inflammatory cell infiltrates in muscle and myositis-specific/associated autoantibodies. It is unclear which pathways are involved in IIM, and the functional relationship between autoantibody targets has not been systematically explored. Protein-protein interaction and pathway analyses were conducted to identify pathways relevant to disease, using autoantibody targets and gene products of IIM-associated single nucleotide polymorphism (SNP) loci.

**Methods:**

Protein-protein interactions were analysed using Disease Association Protein-Protein Link Evaluator (DAPPLE). Gene ontology and pathway analyses were conducted using Database for Annotation Visualisation and Integrated Discovery (DAVID) and Gene Relationships Across Implicated Loci (GRAIL). Analyses were undertaken including the targets of published autoantibodies, significant and suggestive SNPs from an IIM association study and autoantibody targets plus SNPs combined.

**Results:**

The protein-protein interaction networks formed by autoantibody targets and associated SNPs showed significant direct and/or indirect connectivity (*p* < 0.05). Autoantibody targets plus associated SNPs combined resulted in more significant indirect and common interactor connectivity, suggesting autoantibody targets and proteins encoded by IIM-associated loci may be involved in common pathways. Tumour necrosis factor receptor-associated factor 6 (TRAF6) was identified as a hub protein, and *UBE3B, HSPA1A, HSPA1B* and *PSMD3* also were identified as genes with significant connectivity. Pathway analysis identified that autoantibody targets and associated SNP regions are significantly interconnected (*p* < 0.01), and confirmed autoantibody target involvement in translational and post-translational processes. ‘Ubiquitin’ was the only keyword strongly linking significant genes across regions in all three GRAIL analyses of autoantibody targets and IIM-associated SNPs.

**Conclusions:**

Autoantibody targets and IIM-associated loci show significant connectivity and inter-relatedness, and identify several key genes and pathways in IIM pathogenesis, possibly mediated via the ubiquitination pathway.

**Electronic supplementary material:**

The online version of this article (doi:10.1186/s13075-016-1061-7) contains supplementary material, which is available to authorized users.

## Background

The idiopathic inflammatory myopathies (IIM) are a group of rare autoimmune diseases whereby patients typically present with weakness and inflammation of skeletal muscle, and extra-muscular manifestations including interstitial lung disease, malignancy and skin rashes [1]. Approximately 80 % of patients with IIM possess serum autoantibodies; these may be myositis-specific autoantibodies (MSAs), which are found predominantly in IIM, or myositis-associated autoantibodies (MAAs), which are found also in other connective tissue diseases (CTD) or as part of a myositis/CTD-overlap condition. MSAs are associated with a particular clinical profile and are usually mutually exclusive [1].

The most common MSA is anti-Jo-1 which is found in about 20 % of patients with IIM. This MSA targets histidyl tRNA synthetase, one of eight aminoacyl tRNA synthetases (ARS) known to act as autoantigens for MSAs [1]. Anti-ARS are associated with a distinct phenotype known as antisynthetase syndrome, which involves myopathy, non-erosive arthritis, interstitial lung disease, mechanic’s hands and fever [2].

Modification of the structure of an autoantigen, for example, by somatic mutation or post-translational modification, in a pro-immune context such as muscle injury may result in an autoimmune response [2]. In IIM, regenerating muscle cells produce higher levels of autoantigens such as ARS molecules [3]. There is some evidence that ARS molecules may act as chemoattractants inducing migration of immune cells to the affected muscle [4]. Alongside elevated expression of major histocompatibility complex (MHC)-class I on cell surfaces, this may lead to a specific autoimmune response resulting in further muscle damage and regeneration; thereby more autoantigen is produced, resulting in a sustained immune response, disease propagation and amplification [2].

There are currently no disease-specific treatments for IIM; instead anti-inflammatory, immunosuppressive and immunodulatory treatments are borrowed from other rheumatic diseases. Furthermore, despite treatment some patients develop progressive muscle weakness and extramuscular manifestions with ensuing disability and poor quality of life. There is thus a clear need for new targeted treatments in IIM. It is currently unclear which pathways are involved in IIM and whether autoantibodies contribute to the disease process. The functional relationship between autoantibody targets has also not been systematically explored. Protein-protein interaction (PPI) analyses have been performed in a number of complex diseases, including breast cancer, schizophrenia and multiple sclerosis [5–8]. Genome-wide association studies (GWAS) identify single nucleotide polymorphisms (SNPs) associated with particular traits or diseases and PPI analyses have been used to identify the genes most likely to be functionally significant to pathological change [6, 7]; the hypothesis is that associated genes will be involved in a common set of biological pathways or processes, which may be perturbed in the disease. It is possible that the targeting of autoantigens in an autoimmune disease setting like IIM also could be related to these altered pathways.

In this study we analysed PPI and pathways using autoantibody targets and proteins encoded by loci associated with IIM in the largest genetic association study to date [9], to identify pathways involved in IIM. This is the first time a systematic PPI and pathway analysis has been performed in IIM and suggests particular proteins and pathways that may have important roles in disease.

## Methods

### Protein-protein interaction analysis

Analysis was performed using Disease Association Protein-Protein Link Evaluator (DAPPLE) to investigate physical connections between proteins [10]. DAPPLE searches the InWeb database for PPI that have been reported in the literature and assigned a probabilistic score. The InWeb database compiles PPI data from numerous sources including Reactome, IntAct, the Molecular Interaction Database (MINT), the Biomolecular Interaction Network Database (BIND) and the Kyoto Encyclopaedia of Genes and Genomes (KEGG) [10]. DAPPLE is designed to analyse disease-associated single nucleotide polymorphisms (SNPs) on the basis that disease-causing genetic variation is likely to affect common pathways that may be revealed by PPI [10].

**Fig. 1:**
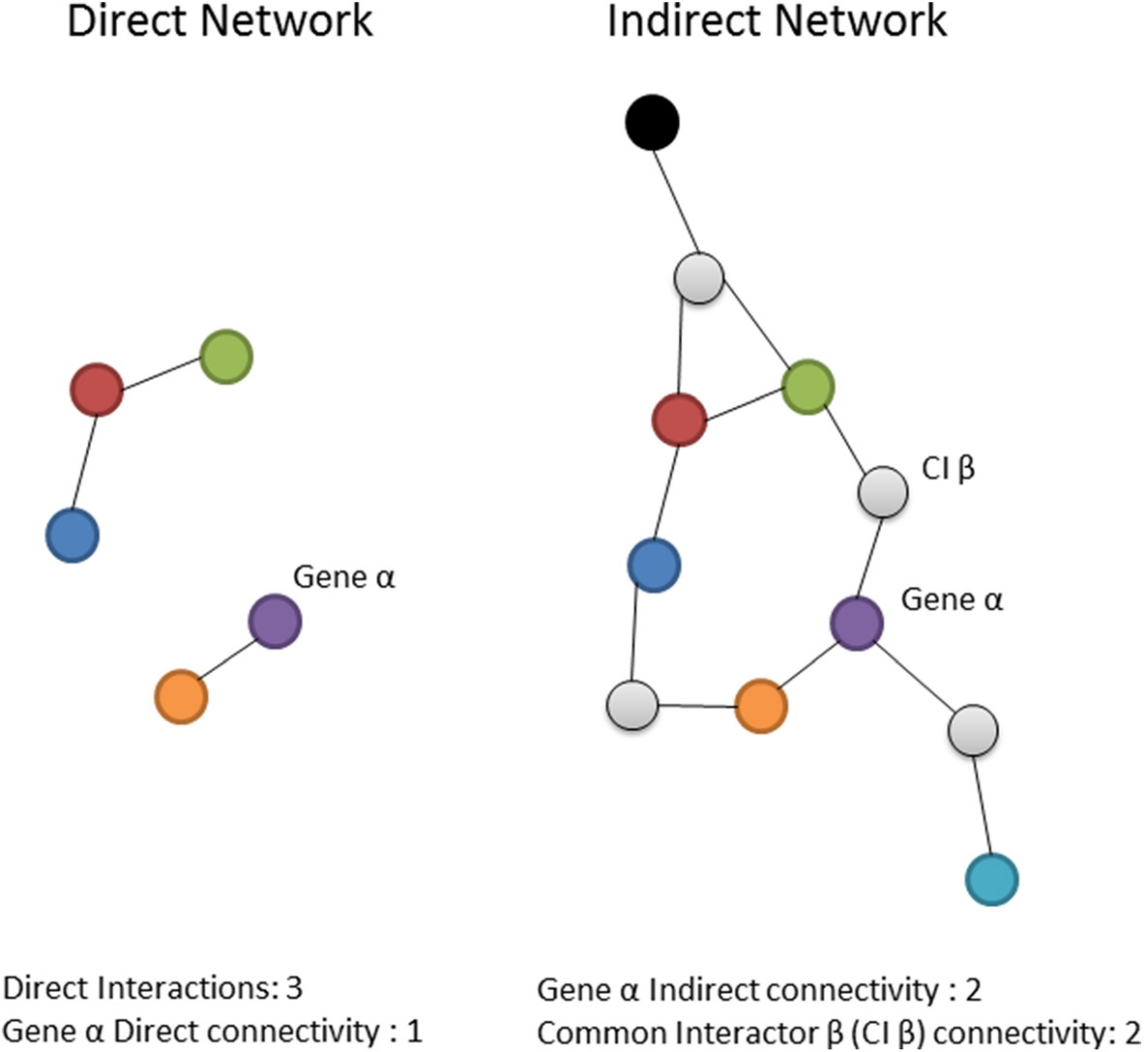
Parameters used to evaluate direct and indirect networks produced by the Disease Association Protein-Protein Link Evaluator (DAPPLE). Protein products of genes in associated loci are shown in *colour* and common interactor proteins in *grey*

Based on these interactions, DAPPLE forms networks of physical protein-protein connectivity where proteins are nodes connected by edges that represent interactions in the InWeb database. A direct connection may be present between two seed proteins (proteins derived from the input SNPs or genes, also referred to as ‘associated proteins’) or an indirect connection may be formed via a common interactor protein (a protein not derived from the input but which interacts with two or more seed proteins). Networks between proteins are evaluated based on four parameters: (1) number of direct interactions (number of edges in the direct network); (2) mean associated protein direct connectivity (the average number of proteins with which each seed protein directly interacts); (3) mean associated protein indirect connectivity (the average number of proteins with which each seed protein indirectly interacts); (4) mean common interactor connectivity (the average number of seed proteins bound by common interactor proteins) (Fig. 1).

The statistical significance of each parameter is assessed using 20,000 permutations, comparing the network formed against networks created by randomly reassigning proteins of the same binding degree as the protein in the original network to each node (binding degree was defined as the total number of interactions a protein has according to the InWeb database). In a random list of proteins any connectivity could simply be a function of the binding degree of each protein. This method controls for bias introduced by the differing extent to which certain PPI have been studied or behave in vitro.

DAPPLE accepts named genes or SNPs as input. From SNP input genes are identified as those in linkage disequilibrium (LD) (*r*^2^ > 0.5), extended to the closest recombination hotspots plus 50 kb. Protein products of genes in associated loci are then scored based on their participation in direct and indirect networks. These scores are Bonferroni corrected for two tests if a protein participates in both networks and further Bonferroni corrected for the number of possible candidates in that locus to identify the ‘genes to prioritise’ (*p*_corr_ < 0.05). [10]. Analysis was performed using DAPPLE, version 2, Hg18, and HapMap SNPs.

### Pathway analyses

Parallel pathway and gene ontology analyses were conducted using Gene Relationships Across Implicated Loci (GRAIL) [11] and the Database for Annotation, Visualisation and Integrated Discovery (DAVID) [12, 13]. GRAIL uses an automated text-based strategy to identify significantly inter-related genes based on a list of disease- associated regions, and identifies the most likely candidate gene in each region [11]. Candidate genes within each region are selected based on a significance score for their relationships to genes in other associated regions, corrected for multiple hypothesis testing for the number of genes in the region. Candidate genes with uncorrected GRAIL text scores (*p*_text_ < 0.2) are used to identify the top twenty highest scoring keywords based on the average frequency of that term for candidate genes versus all genes. These keywords must appear in at least 500 documents, be four or more letters long and contain no numbers [11]. GRAIL makes no assumptions about the phenotype being studied or underlying pathways presumed to be relevant to the disease. Analysis was conducted using gene size correction and the October 2014 text database.

DAVID analysis was conducted using biological processes from the Gene ontology consortium (GOTERM_BP_FAT) and pathways from KEGG using DAVID version 6.7. *P* values were Benjamini-Hochberg corrected for multiple hypothesis testing.

### Input for protein-protein interaction and pathway analyses

Analyses were performed using the following inputs: (1) all published MSA targets and anti-PMScl-75/100 targets; (2) the most significantly associated SNPs from each region from a recently reported IIM Immunochip association study [9]; (3) the combination of SNPs and autoantibody targets (Table 1; these data are also available in Additional file 1). Anti-PMScl-75/100 was included despite being classified as an MAA, as it is almost always mutually exclusive to other MSA/MAAs.

**Table 1:** Myositis-specific and associated autoantibody targets and significant or suggestive SNPs used in analyses

Autoantibody targets (*n* = 28)			SNPs (*n* = 22)			
Symbol	Description	MSA/MAA	rsID	Coordinates (GRCh37 assembly)	Immunochip *p* value [9]	Clinical group
**GARS**	**Glycyl-tRNA synthetase**	**Anti-EJ**	**rs5754467**	Chr22:21985094	4.67 × 10^-07^	IIM
**YARS**	**Tyrosyl-tRNA synthetase**	**Anti-Ha**	**rs6599390**	Chr4:956047	6.48 × 10^-07^	IIM
**HARS**	**Histidyl-tRNA synthetase**	**Anti-Jo-1**	**rs4853540**	Chr2:191917317	1.57 × 10^-06^	IIM
**NARS**	**Asparaginyl-tRNA synthetase**	**Anti-KS**	**rs10189330**	Chr2:99389870	2.68 × 10^-06^	IIM
**IARS**	**Isoleucyl-tRNA synthetase**	**Anti-OJ**	**rs570676**	Chr11:36492191	9.42 × 10^-06^	IIM
**AARS**	**Alanyl-tRNA synthetase**	**Anti-PL-12**	**rs223900**	Chr16:57445376	9.97 × 10^-06^	IIM
**TARS**	**Threonyl-tRNA synthetase**	**Anti-PL-7**	**rs451375** (Proxy for rs376072)	Chr3:28049889	(1.45 × 10^-05^)	IIM
FARSA	Phenylalanyl-tRNA synthetase, alpha subunit	Anti-Zo-α	**rs3116494**	Chr2:204592021	1.54 × 10^-05^	IIM
**FARSB**	**Phenylalanyl-tRNA synthetase, beta subunit**	**Anti-Zo-β**	**rs11064180**	Chr12:6523249	1.61 × 10^-05^	IIM
CHD3	Chromodomain helicase DNA binding protein 3	Anti-Mi-2α	**rs2476601**	Chr1:114377568	7.22 × 10^-09^/7.90 × 10^-11^	IIM/PM
**CHD4** ^**a**^	**Chromodomain helicase DNA binding protein 4**	**Anti-Mi-2β**	rs3094013	Chr6:31434366	6.36 × 10^-76^	PM
**SRP54**	**Signal recognition particle 54 kDa**	**Anti-SRP**	**rs9905921**	Chr17:71527243	2.01 × 10^-06^	PM
SAE1	Small ubiquitin related modifier 1 activating enzyme subunit 1	Anti-SAE-1	**rs7956536**	Chr12:109980516	3.66 × 10^-06^	PM
**UBA2**	**Ubiquitin-like modifier activating enzyme 2**	**Anti-SAE-2**	rs2286896	Chr2:191535576	3.76 × 10^-06^	PM
**TRIM33**	**Tripartite motif containing 33**	**Anti-TIF1γ**	**rs17799348**	Chr8:11333521	4.13 × 10^-06^	PM
TRIM24	Tripartite motif containing 24	Anti-TIF1α	**rs917998** (Proxy for rs1420095)	Chr2:103068156	(6.16 × 10^-06^)	PM
TRIM28	Tripartite motif containing 28	Anti-TIF1β	rs4690220 (Proxy rs11724804 in GRAIL)	Chr4:980464	7.47 × 10^-06^	PM
**IFIH1**	**Interferon induced with helicase C domain 1**	**Anti-MDA5**	**rs7535818** (Proxy rs2984920 in GRAIL)	Chr1:192545099	1.37 × 10^-05^	PM
**NT5C1A**	**5'-nucleotidase, cytosolic 1A**	Anti-NT5C1A	**rs3129927**	Chr6:32333827	3.74 × 10^-48^/2.06 × 10^-129^	DM/JDM/IIM
**ABTB1**	**Ankyrin repeat and BTB (POZ) domain containing 1**	**Anti-Fer**	**rs4702698**	Chr5:10517908	4.77 × 10^-06^	DM/JDM
**HMGCR**	**3-hydroxy-3-methylglutaryl-CoA reductase**	**Anti-HMGCR**	**rs4921293**	Chr5:159928876	8.27 × 10^-06^	DM/JDM
**MORC3**	**MORC family CW-type zinc finger 3**	**Anti-NXP2**	**rs1008723**	Chr17:38066267	9.05 × 10^-06^	DM/JDM
**PMS1**	**Postmeiotic segregation increased 1**	**Anti-PMS1**				
PMS2	Postmeiotic segregation increased 2	Anti-PMS2				
**MLH1**	**MutL homolog 1**	**Anti-MLH1**				
**PRKDC**	**Protein kinase, DNA-activated, catalytic polypeptide**	**Anti-DNA PKCS**				
EXOSC9	Exosome component 9	Anti-PMScl-75				
**EXOSC10**	**Exosome component 10**	**Anti-PMScl-100**				

Single nucleotide polymorphisms (SNPs) were included if they reached the suggestive significance threshold (*p* < 2.25 × 10^-5^) for association with idiopathic inflammatory myopathies (*IIM*) in the Immunochip study published by Rothwell et al., 2015. Subset of autoantibody targets used in Disease Association Protein-Protein Link Evaluator (*DAPPLE*) formed by selecting one protein from each complex are shown in bold (*n* = 21). Subset of SNPs used in Gene Relationships Across Implicated Loci (GRAIL) analysis due to overlapping regions also shown in bold (*n* = 19) and proxies used for unrecognised SNPs are in brackets. ^a^CHD4 also not included in GRAIL analysis due to overlapping regions (autoantibody targets = 27). Clinical groups included polymyositis (PM), adult/juvenile dermatomyositis (DM/JDM) and all sub-groups combined (IIM)

The Uniprot IDs of all published MSA and anti-PMScl-75/100 targets were identified (*n* = 28). SNPs reaching GWAS thresholds for significance (*p* < 5 × 10^-8^) or suggestive significance (*p* < 2.25 × 10^-5^, determined using the genetic type I error calculator [14]) in the IIM Immunochip association study [9] were identified from each clinical subgroup (combined IIM, polymyositis, adult/juvenile dermatomyositis) (*n* = 22). The genetic type 1 error calculator estimates the effective number of independent tests based on the LD between SNPs contained on the genotyping array. The most significantly associated SNP was used for each region, or a proxy was used if this SNP was not recognised by DAPPLE (proxy SNP in LD *r*^2^ ≥ 0.9 with associated SNP, identified using SNAP version 2.2, hg18, HapMap 22 [15]). Input genes (encoding proteins that may participate in these networks) were identified based on LD (*r*^2^ > 0.5 with the associated SNP) and extension to the closest recombination hotspot for GRAIL, or extension to the closest recombination hotspot +50 kb for DAPPLE [10], resulting in 185 and 151 genes used as input for GRAIL and DAPPLE, respectively. For DAPPLE the analysis was also run using a subset of autoantibody targets selecting one protein from each complex (*n* = 21), to remove bias from known interactions; this subset was used in the combined autoantibody targets-SNP analysis (*n* = 43). The genes from associated SNP regions identified by DAPPLE were used as input for DAVID; MHC region SNPs were excluded from the DAVID analysis to remove bias introduced by the strong LD and large number of immune-related genes across this region, resulting in an input of 62 genes from 20 SNPs plus the 28 autoantibody targets (*n* = 90).

## Results

### Protein-protein interaction analysis identifies significant direct and indirect connectivity

**Fig. 2:**
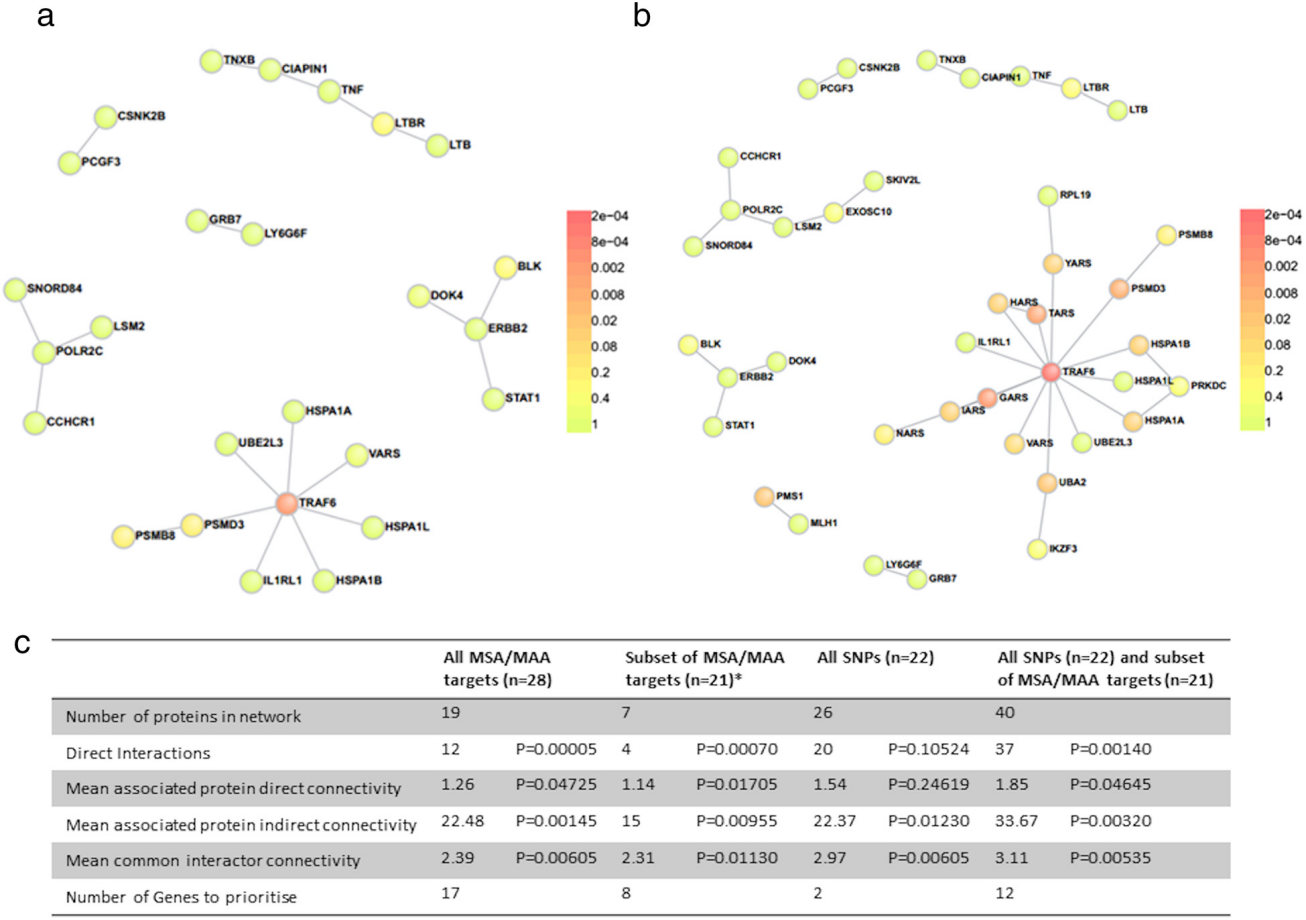
Protein-protein interaction analyses of associated single nucleotide polymorphism (SNP) loci and targets of myositis autoantibodies. **a** Direct network for all SNPs of significant or suggestive significance (*p* < 2.25 × 10^-5^) reported in the idiopathic inflammatory myopathies (IIM) Immunochip study. **b** Direct network for all SNPs of significant or suggestive significance and subset of myositis autoantibody targets (*one protein selected from each complex). *Colours* indicate significance of node *p* value. **c** Summary statistics

The PPI networks formed by autoantibody targets and IIM-associated SNPs using DAPPLE had significant direct and/or indirect connectivity (*p*_corr_ < 0.05, Fig. 2, Additional file 2), including when only one protein was selected from each known autoantibody target complex. These results indicate that these proteins interact more than a group of randomly selected proteins in 20,000 permutations of the networks and therefore could be involved in common molecular networks linked to IIM. Due to strong direct PPI, 17/28 autoantibody targets were identified as genes to prioritize as they participate in the network more than would be expected by chance (*p*_corr_ values <0.05, Table 2). SNP loci alone had significant indirect and common interactor connectivity but direct connectivity was not significant, suggesting that the proteins encoded by associated loci do not directly interact more than a randomly selected group of proteins but may be involved in common pathways (indirect networks and common interactor *p* values may be found in Additional file 3). The SNP analysis identified *UBE3B* (*p*_corr_ = 8.03 × 10^-4^) and *TRAF6* (p_corr_ = 2.11 × 10^-3^) as genes to prioritise. Inclusion of both autoantibody targets and IIM-associated SNPs resulted in greater and more significant indirect and common interactor connectivity than either network alone, suggesting indirect interaction between autoantibody targets and proteins encoded by IIM-associated loci (Fig. 2b and c). Including both SNPs and MSA/MAA targets additionally identified *PSMD3, HSPA1A* and *HSPA1B* alongside *UBE3B* and *TRAF6* and seven myositis-specific autoantibody (MSA) targets as genes to prioritise (Table 2). From visual assessment of the direct networks tumour necrosis factor receptor-associated factor 6 (TRAF6) appears to act as a hub protein, interacting with many proteins for both SNP and SNP-autoantibody target networks (Fig. 2a and b).

**Table 2:** Genes to prioritise from protein-protein interaction analyses of SNPs and targets of myositis-specific antibodies (MSA) or myositis-associated autoantibodies (MAA)

All MSA/MAA targets (*n* = 28)		Subset of MSA/MAA targets^a^ (*n* = 21)		All SNPs (*n* = 22)		All SNPs (*n* = 22) and subsets of MSA/MAA targets^a^ (*n* = 21)	
Gene	*P*_corr_ value	Gene	*P*_corr_ value	Gene	*P*_corr_ value	Gene	*P*_corr_ value
UBA2	3.03 × 10^-4^	GARS	3.16 × 10^-4^	UBE3B	8.03 × 10^-4^	TRAF6	3.01 × 10^-4^
GARS	5.05 × 10^-4^	PMS1	4.21 × 10^-4^	TRAF6	2.11 × 10^-3^	UBE3B	4.01 × 10^-4^
PMS1	3.63 × 10^-3^	UBA2	3.47 × 10^-3^			GARS	1.20 × 10^-3^
TRIM24	4.74 × 10^-3^	IARS	5.68 × 10^-3^			TARS	3.90 × 10^-3^
YARS	8.37 × 10^-3^	YARS	1.09 × 10^-2^			PSMD3	6.99 × 10^-3^
TRIM28	8.97 × 10^-3^	HARS	1.15 × 10^-2^			UBA2	1.19 × 10^-2^
NARS	9.17 × 10^-3^	AARS	1.78 × 10^-2^			PMS1	1.67 × 10^-2^
TRIM33	1.10 × 10^-2^	NARS	3.23 × 10^-2^			HSPA1B	2.73 × 10^-2^
IARS	1.15 × 10^-2^					YARS	3.79 × 10^-2^
MLH1	1.79 × 10^-2^					IARS	3.98 × 10^-2^
HARS	1.95 × 10^-2^					HARS	4.61 × 10^-2^
CHD3	2.67 × 10^-2^					HSPA1A	4.94 × 10^-2^
FARSA	3.02 × 10^-2^						
AARS	3.10 × 10^-2^						
TARS	3.23 × 10^-2^						
EXOSC9	3.43 × 10^-2^						
FARSB	4.61 × 10^-2^						

Listed in order of significance from most to least significant. *P*_cor_ -values corrected for multiple testing. ^a^Subset of autoantibody targets formed by selecting one protein from each complex.

### Text-based pathway analysis of autoantibody targets and associated SNP loci identifies significant inter-relatedness

GRAIL analysis identified 24/28 autoantibody targets as significant based on their connections to the other autoantibody targets in the literature (*p*_corr_ < 0.01 for each autoantibody target). Only PRKDC, ABTB1, HMGCR and NT5C1A were not significant (Additional file 4). The twenty keywords identified by GRAIL confirmed previous understanding of MSA target functions (‘tRNA’, ‘synthetase’) and suggested autoantibody target involvement in post-translational modification (‘sumoylation’, ‘ubiquitin’) (Additional file 5). The VIZ-GRAIL (Visualising GRAIL connections) plot showed strong connections (*p* < 0.05), especially between the anti-synthetase targets and within known complexes such as CHD3-CHD4 and SAE1-UBA2 (Additional file 6).

**Fig. 3:**
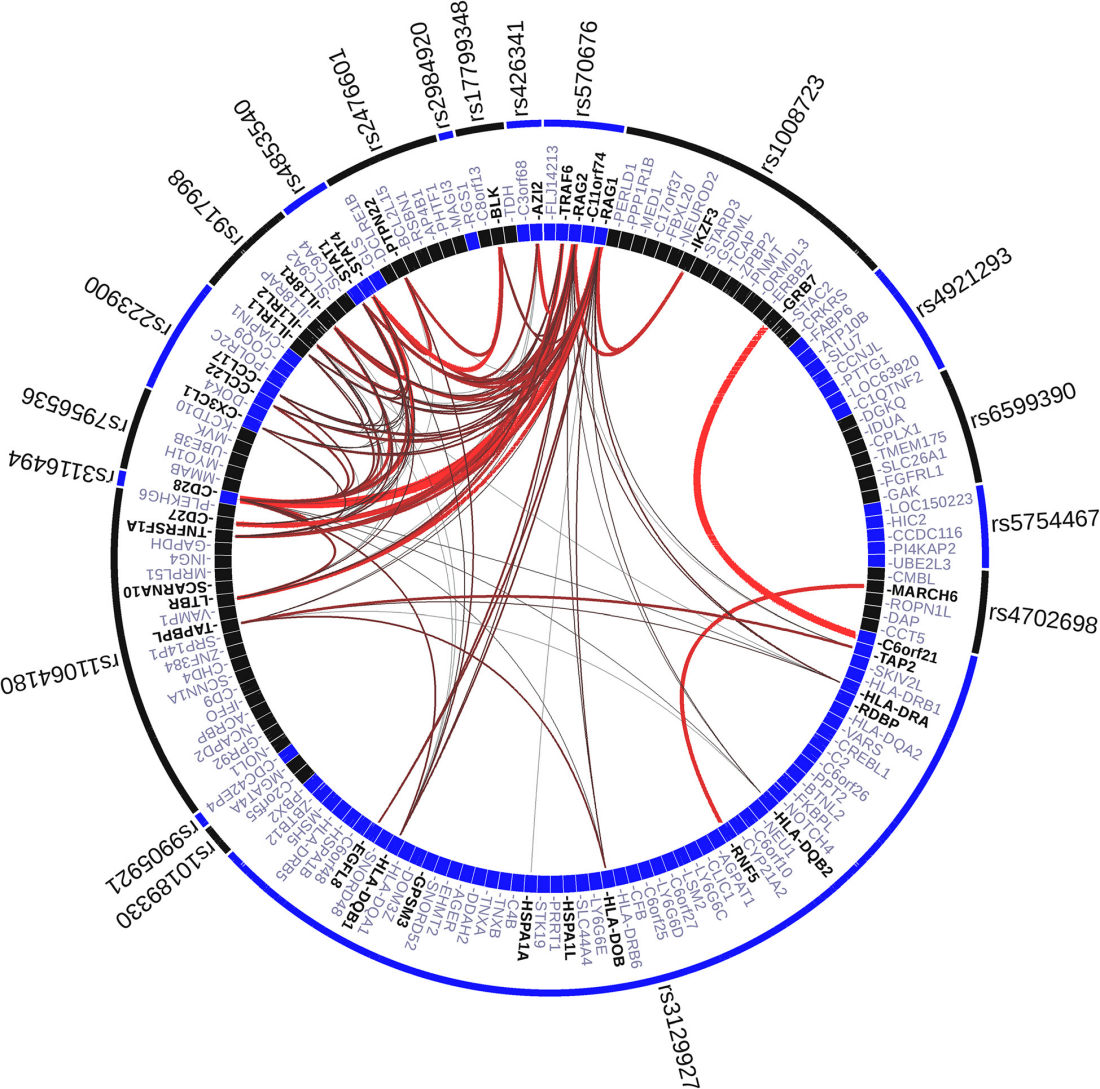
Visualising Gene Relationships Across Implicated Loci (VIZ-GRAIL) plot of 19 associated single nucleotide polymorphism (SNP) loci. *Outer circle* labelled with SNPs, *inner circle* with candidate genes. *Thickness* and *brightness* of *lines* represents the strength of the connections between genes, VIZ-GRAIL (Visualising GRAIL connections)

In analysis of SNPs, only 4/19 regions were identified as significant based on their connections to the other associated regions (*p*_corr_ < 0.01 for each candidate gene); the most likely candidate genes *STAT4, CD27, CCL17* and *CD28* (Additional file 4). The majority of keywords identified were immune-related, but also included ‘ubiquitin’ (Additional file 5). The VIZ-GRAIL plot showed many strong pairwise connections between candidate genes from associated loci such as *C6orf21-GRB7, BLK-STAT4, CD27-RAG2* and *CD28-RAG1* (Fig. 3).

GRAIL analysis of SNPs and autoantibody targets combined identified an increased number of autoantibody targets (25/27) and candidate genes (12/19) as significantly interconnected to the other associated SNP regions or autoantibody targets (*p*_corr_ < 0.01 for each autoantibody target or candidate gene), suggesting inter-relatedness between autoantibody targets and gene products of IIM-associated loci (Additional files 4 and 7). Notably, *STAT1* was identified as the most likely candidate gene in the combined analysis from the rs4853540 association, rather than *STAT4*. Keywords included ‘ubiquitin’ and ‘sumoylation’ and immune and tRNA synthetase-related terms (Additional file 6).

### Gene ontology analyses confirm known pathways and suggest novel pathways involved in IIM pathogenesis

DAVID gene ontology analysis of 28 autoantibody targets confirmed previous knowledge of MSA target involvement in translation and RNA metabolism, including amino acid activation (*p*_corr_ = 7.6 × 10^-12^), tRNA aminoacylation (*p*_corr_ = 7.6 × 10^-12^), tRNA metabolic process (*p*_corr_ = 6.5 × 10^-9^), non-coding RNA metabolic process (*p*_corr_ = 2.1 × 10^-9^) and translation (*p*_corr_ = 8.5 × 10^-7^) (Additional files 8 and 9). Autoantibody targets were shown also to be significantly involved in somatic recombination of immunoglobulin gene segments (*p*_corr_ = 4.3 × 10^-2^). The only significant KEGG pathway was aminoacyl-tRNA biosynthesis (*p*_corr_ = 9.8 × 10^-12^), which is unsurprising as nine of the autoantibody targets are aminoacyl tRNA synthetases. Gene ontology analysis of the associated SNP loci identified only immune response as significant (*p*_corr_ = 0.015). Combined analyses of autoantibody targets and associated SNP loci identified additional significant immune-related gene ontologies such as somatic diversification of immune receptors (*p*_corr_ = 2.9 × 10^-3^).

## Discussion

We report the first systematic protein-protein interaction and pathway analysis of myositis autoantibody targets and proteins encoded by IIM-associated loci [9]. Our analyses identify significant direct and indirect connectivity and inter-related genes/proteins. Several genes are highlighted by PPI analysis including *TRAF6, HSPA1A/B, UBE3B* and *PSMD3*. Pathway analysis confirmed the over-representation of autoantibody target involvement in translation and suggests that ubiquitination may play an important role in the IIM disease processes.

The significant direct and indirect connectivity identified between autoantibody targets indicates physical interactions between these antigens and with common interactors and suggests related biological pathways. The significant indirect connectivity between associated SNP loci suggests that although proteins encoded by these loci do not directly interact, they may influence common pathways, in keeping with potential long-range regulatory effects. The more significant indirect and common interactor connectivity observed in the combined analysis suggests autoantibody targets and proteins encoded by IIM-associated loci act in the same pathways.

The direct networks produced by DAPPLE suggest an important role for TRAF6 interactions with both autoantibody targets and SNP-associated gene products in IIM. TRAF6 is an E3 ligase which acts as an intermediate signalling adaptor between signals such as pathogen-associated molecular patterns (PAMPs) and downstream pathways including nuclear factor-kB (NF-kb), c-Jun N-terminal kinase (JNK), mitogen-activated protein kinase (MAPK) and AMP-activated protein kinase (AMPK). TRAF6 is involved in immune responses via the regulation of inflammation [16], differentiation and proliferation of B cells [17] and development of medullary thymic epithelial cells [18]. TRAF6 also is implicated in muscle atrophy [19]. These roles support the hypothesis that TRAF6 may have an important role within muscle as part of the autoimmune, inflammatory process in IIM, and TRAF6 with associated pathways may represent potential therapeutic targets.

Of the seven autoantibody target-derived ‘genes to prioritise’ in the DAPPLE analysis of SNPs and autoantibody targets, five were aminoacyl tRNA synthetases (ARS). In the direct network four of these (TARS, HARS, GARS and YARS) directly interacted with TRAF6. Some of the statistical significance of these proteins may be derived from interactions within the ARS group. However, a proportion will be due to their interaction with TRAF6. Further study of these interactions may be valuable in understanding the link between presence of anti-ARS and the specific phenotype observed in anti-synthetase syndrome.

**Fig. 4:**
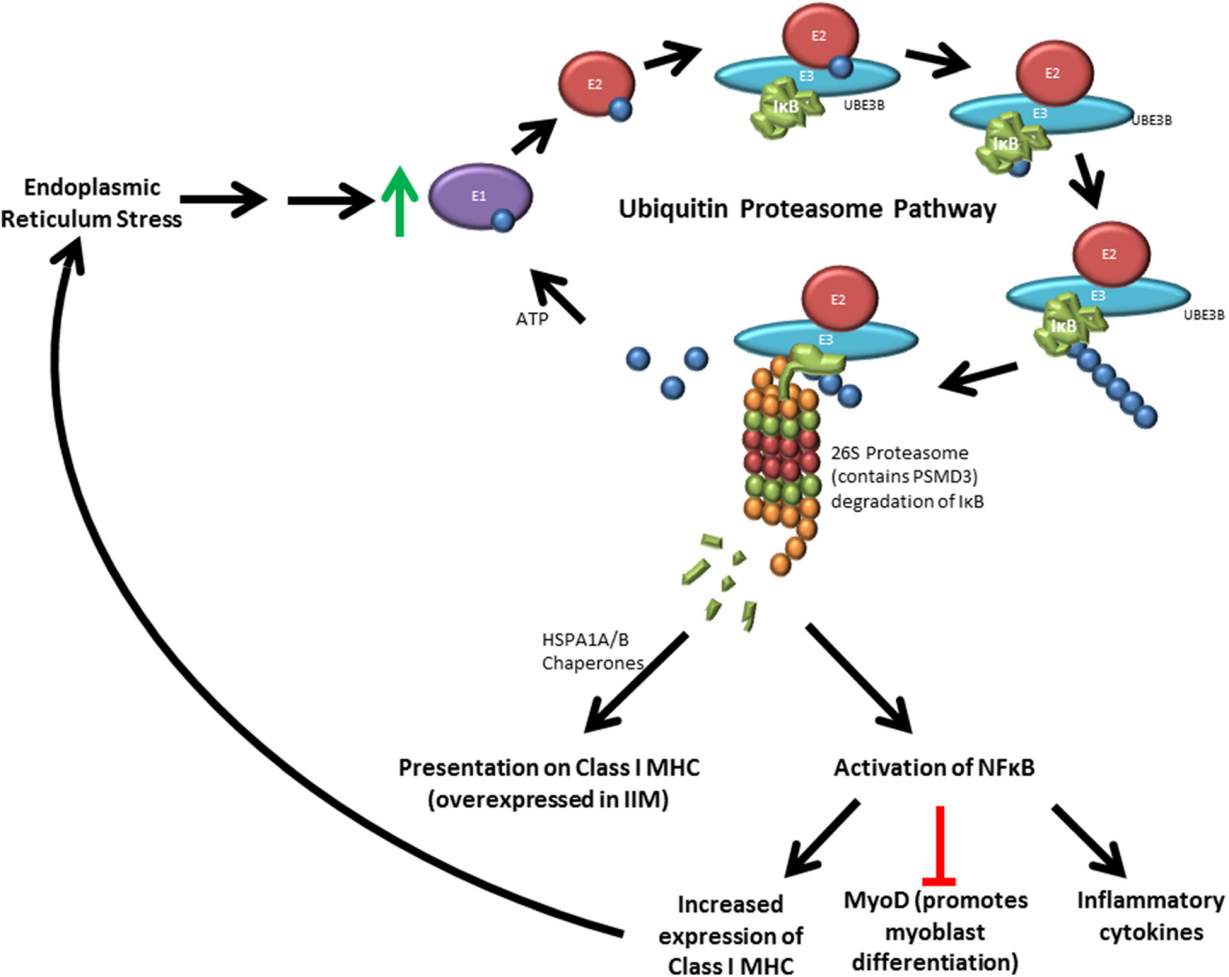
Proposed ubiquitin proteasome pathway (UPP) involvement in idiopathic inflammatory myopathies (IIM). Endoplasmic reticulum (ER) stress leads to upregulation of the UPP. Ubiquitin binds to the E1 ubiquitin activating enzyme, which is then replaced by E2 ubiquitin conjugating enzyme. E3 ubiquitin ligase then brings in the target protein, in this example, inhibitor of nuclear factor kB (IkB), and catalyses repeated ligation to the ubiquitin. UBE3B is an E3 ligase. The polyubiquitinated protein is targeted to the 26S proteasome (of which PSMD3 is a component) and degraded. Peptide fragments can then be chaperoned by proteins such as HSPA1A/B to major histocompatibility complex (*MHC*) class I for presentation. The degradation of IkB results in activation of NFkB, which promotes production of MHC class I and inflammatory cytokines and inhibits MyoD. MHC class I overexpression results in further ER stress, MyoD inhibition results in reduced myoblast differentiation and inflammatory cytokines cause damage to muscle fibres

A further ‘gene to prioritise’, *UBE3B,* is not present in the direct networks, and derives significant connectivity indirectly via common interactor proteins. *UBE3B* encodes ubiquitin protein ligase E3B, which is involved in target recognition in the ubiquitination pathway. Another ‘gene to prioritise’, *PSMD3*, encodes a 26 s proteasome regulatory subunit involved in degradation of ubiquitinated proteins via the ubiquitin proteasome pathway (UPP). In the Immunochip study two associations were reported as intergenic of *PRR5L/TRAF6* and *UBE3B/MMAB* [9]. Our analyses could suggest *TRAF6* and *UBE3B* as the causal genes in their respective regions. *UBE2L3*, an E2 ubiquitin-conjugating enzyme, was also highlighted in the Immunochip study as it has previously been implicated in other autoimmune diseases. Four of five prioritised SNP-derived genes identified in the DAPPLE analyses encode proteins related to the UPP (Fig. 4). Furthermore, ‘ubiquitin’ was the only keyword identified in all three GRAIL analyses (Additional file 6). In IIM, ubiquitination is involved in activation of NF-kB which in turn leads to upregulation of MHC class I, promotion of inflammatory cytokines leading to muscle fibre damage, and MyoD inhibition resulting in reduced myoblast differentiation (Fig. 4) [20].

The UPP is thought to be the most significant degradation system in myogenesis [21]. Inhibition or knockdown of the proteasome prevents myoblast fusion and inhibits differentiation, whereas proteasomal activity increases during differentiation of mouse myoblast C212 cells [21] and has been found to be increased in IIM muscle tissue [22].

In inclusion body myositis (IBM), abnormalities of muscle protein homeostasis may lead to accumulation of proteins in muscle fibres, possibly as a result of impaired proteasome function. Gene mutations resulting in disrupted ubiquitination have been identified in several inherited myopathies such as nemaline and polyglucosan body myopathy [23, 24].

As illustrated by Fig. 4, enhanced proteasome activity generates MHC class I restricted antigens, leading to activation of the immune response. Stress-inducible heat shock 70 kDa (Hsp70) proteins, such as HSPA1A and HSPA1B, upregulated when cells are under stress, act as chaperones for transferring these antigens from the proteasome to be presented by MHC class I or II [25]. Hsp70 proteins play a critical role in protecting against muscle damage in the innate immune response, promoting muscle regeneration and recovery, and maintaining skeletal muscle mass and integrity [26]. This response decreases with increased age. As IIM is generally a disease of middle and old age it is thought that restoration of the heat-shock response may be a good therapeutic approach, especially in IBM where protein mishandling is most clearly seen in the form of protein aggregates in muscle fibres. Arimoclomol is a pharmacological agent that induces an increased heat-shock response in stressed cells but does not appear to affect unstressed cells, thus avoiding off-target responses [27]. Arimoclomol has shown promise in ameliorating disease in both in vitro rat myoblast and in vivo mouse models of IBM and has passed safety testing in IBM patients [27]. A further pharmacological agent, 17-N-allylamino-17-demethoxygeldanamycin (17AAG), upregulates Hsp70. Treatment with 17AAG has been shown to preserve mitochondrial function and prevent atrophy of C2C12 mouse myotubes in a tunicamycin-induced endoplasmic reticulum (ER)-stress model of IIM [28]. This PPI analysis highlighting heat-shock proteins in association with IIM lends further support to targeting the heat shock response in development of future IIM treatments.

A limitation of this study is that MSA/MAAs target intracellular antigens and it is unclear whether the autoantibodies or the pathways in which the antigens are involved influence the pathogenesis of IIM, and how these antigens come to be exposed to the immune system in IIM break of tolerance. The IIM-associated SNPs are from an Immunochip study, therefore, the identification of immune-related keywords is likely influenced by the biased content of the Immunochip. However, these results are still informative and highlight non-immune pathways that could be investigated as potential therapeutic targets in IIM.

## Conclusions

In conclusion, through PPI and pathway analyses, this study highlights several genes including *TRAF6, HSPA1A/B, UBE3B* and *PSMD3*, and pathways including ubiquitination, which may play important roles in IIM disease processes.

## Abbreviations

ARS, aminoacyl tRNA synthetase; BIND, Biomolecular Interaction Network Database; CTD, connective tissue disease; DAPPLE, Disease Association Protein-Protein Link Evaluator; DAVID, Database for Annotation Visualisation and Integrated Discovery; ER, endoplasmic reticulum; GRAIL, Gene Relationships Across Implicated Loci; GWAS, genome-wide association studies; IBM, inclusion body myositis; IIM, idiopathic inflammatory myopathies; KEGG, Kyoto Encyclopaedia of Genes and Genomes; LD, linkage disequilibrium; MAA, myositis-associated autoantibody; MHC, major histocompatibility complex; MINT, Molecular Interaction Database; MSA, myositis-specific autoantibody; PAMPs, Pathogen associated molecular patterns; PPI, protein-protein interactions; SNP, single nucleotide polymorphism; TRAF, tumour necrosis factor receptor-associated factor; UPP, ubiquitin proteasome pathway.

## Additional files

Additional file 1: Input for analyses. Excel file of the information in Table 1 about myositis-specific and associated autoantibody targets and significant or suggestive SNPs used in analyses (XLSX 12 kb) Additional file 2: Figure S1. Protein-protein interaction analysis showing direct networks among myositis autoantibody targets. **a** Myositis autoantibody targets. **b** Subset of myositis autoantibody targets (one selected from each complex). *Colours* indicate significance of node *p* value (TIF 97 kb) Additional file 3: DAPPLE output. Excel file of the output provided by http://www.broadinstitute.org/mpg/dapple/dappleTMP.php from each DAPPLE analysis on SNPs, autoantibody targets, a subset of autoantibody targets and SNPs and autoantibody target subset combined (XLSX 6162 kb) Additional file 4: Table S1. Most likely candidate genes in each significantly interconnected region identified by text-based pathway analysis, using GRAIL. Inputs were 19 myositis-associated SNPs, 28 autoantibody targets and 19 SNPs and 27 autoantibody targets combined. Due to the same candidate genes being selected from multiple inputs, CHD4, rs2286896, rs11724804 and rs3094013 were removed from the analysis. *NS* not significant (*p* > 0.01), *ND* not determined (DOCX 23 kb) Additional file 5: Table S2. Keywords strongly linking the significant genes in each region identified by text-based pathway analysis, using GRAIL. Inputs were 19 myositis-associated SNPs, 28 autoantibody targets and 19 SNPs and 27 auotantibody targets combined. Due to the same candidate genes being selected from multiple inputs, CHD4, rs2286896, rs11724804 and rs3094013 were removed from the analysis (DOCX 15.1 KB) Additional file 6: Figure S2. VIZ-GRAIL plot of 28 myositis autoantibody targets. *Outer circle* labelled with regions, *inner circle* with genes. *Thickness* and *brightness* of *lines* represents the strength of the connections between genes (TIF 672 kb) Additional file 7: Figure S3. VIZ-GRAIL plot of 27 myositis autoantibody targets and 19 SNPs. *Outer circle* labelled with regions/SNPs, *inner circle* with genes. *Thickness* and *brightness* of *lines* represents the strength of the connections between genes (TIF 958 kb) Additional file 8: Table S3. Significant DAVID results for gene ontology and pathway analysis of autoantibody targets and genes from associated loci. Genes were nominated by DAPPLE for 20 associated SNPs (the two MHC SNPs were excluded). Only significant results are shown (Benjamini-Hochberg false discovery rate <0.05) (DOCX 16 kb) Additional file 9: DAVID output. Excel file of the output provided by https://david.ncifcrf.gov/from each DAPPLE analysis on SNPs, autoantibody targets, a subset of autoantibody targets and SNPs and autoantibody target subset combined (XLSX 25 kb)
