# Supplementary figures and images for: Systematic protein-protein interaction and pathway analyses in the idiopathic inflammatory myopathies
Source: Arthritis Res Ther. 2016 Jul 7;18:156. doi: 10.1186/s13075-016-1061-7 (PMC4936183; doi:10.1186/s13075-016-1061-7)

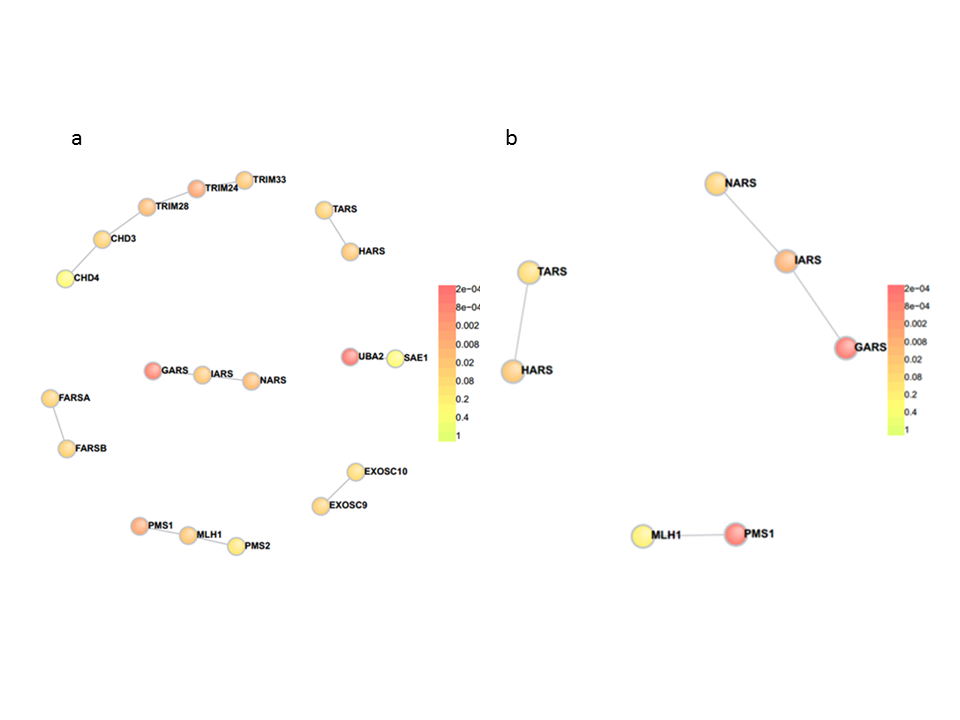

Supplement: Additional file 2: Figure S1. — Protein-protein interaction analysis showing direct networks among myositis autoantibody targets. a Myositis autoantibody targets. b Subset of myositis autoantibody targets (one selected from each complex). Colours indicate significance of node p value (TIF 97 kb) [file 13075_2016_1061_MOESM2_ESM.tif]
