# Supplementary material for: Systematic protein-protein interaction and pathway analyses in the idiopathic inflammatory myopathies
Source: Arthritis Res Ther. 2016 Jul 7;18:156. doi: 10.1186/s13075-016-1061-7 (PMC4936183; doi:10.1186/s13075-016-1061-7)
Supplement: Additional file 4: Table S1. — Most likely candidate genes in each significantly interconnected region identified by text-based pathway analysis, using GRAIL. Inputs were 19 myositis-associated SNPs, 28 autoantibody targets and 19 SNPs and 27 autoantibody targets combined. Due to the same candidate genes being selected from multiple inputs, CHD4, rs2286896, rs11724804 and rs3094013 were removed from the analysis. NS not significant (p > 0.01), ND not determined (DOCX 23 kb) [file 13075_2016_1061_MOESM4_ESM.docx]

**Table S1. Most likely candidate genes in each significantly interconnected region identified by text-based pathway analysis, using GRAIL.**

|  | **28 MSA/MAAs** | **19 SNPs** | | **19 SNPs and 27 MSA/MAAs** | |
| --- | --- | --- | --- | --- | --- |
| **Region** | **GRAIL p-value** | **GRAIL p-value** | **Candidate gene** | **GRAIL p-value** | **Candidate gene** |
| **TARS** | 7.62E-14 |  |  | 4.96E-13 |  |
| **IARS** | 1.86E-13 |  |  | 8.06E-13 |  |
| **HARS** | 1.00E-13 |  |  | 1.43E-12 |  |
| **GARS** | 1.24E-13 |  |  | 2.25E-12 |  |
| **NARS** | 4.55E-13 |  |  | 4.06E-12 |  |
| **AARS** | 1.15E-12 |  |  | 1.75E-11 |  |
| **FARSB** | 6.61E-12 |  |  | 6.33E-11 |  |
| **YARS** | 3.88E-12 |  |  | 7.75E-11 |  |
| **rs3129927** |  | NS |  | 1.47E-10 | VARS |
| **MORC3** | 4.21E-11 |  |  | 1.71E-10 |  |
| **FARSA** | 7.88E-10 |  |  | 4.24E-09 |  |
| **EXOSC10** | 1.78E-08 |  |  | 1.57E-08 |  |
| **EXOSC9** | 7.62E-06 |  |  | 6.57E-06 |  |
| **TRIM33** | 1.65E-06 |  |  | 6.86E-06 |  |
| **SAE1** | 2.35E-06 |  |  | 7.60E-06 |  |
| **UBA2** | 2.96E-06 |  |  | 8.79E-06 |  |
| **TRIM28** | 2.40E-07 |  |  | 1.35E-05 |  |
| **TRIM24** | 3.94E-06 |  |  | 2.45E-05 |  |
| **IFIH1** | 9.60E-03 |  |  | 2.99E-05 |  |
| **CHD3** | 1.69E-06 |  |  | 3.90E-05 |  |
| **rs4853540** |  | 2.60E-04 | STAT4 | 4.34E-05 | STAT1 |
| **PMS2** | 1.98E-05 |  |  | 5.79E-05 |  |
| **rs11064180** |  | 4.50E-04 | CD27 | 1.97E-04 | CD27 |
| **rs426341** |  | NS |  | 1.99E-04 | AZI2 |
| **PRKDC** | NS |  |  | 2.45E-04 |  |
| **PMS1** | 7.39E-05 |  |  | 3.10E-04 |  |
| **rs223900** |  | 6.30E-04 | CCL17 | 3.92E-04 | CCL17 |
| **rs17799348** |  | NS |  | 6.01E-04 | TDH |
| **rs3116494** |  | 2.76E-03 | CD28 | 8.08E-04 | CD28 |
| **rs570676** |  | NS |  | 9.18E-04 | RAG1 |
| **MLH1** | 1.75E-04 |  |  | 1.30E-03 |  |
| **rs1008723** |  | NS |  | 1.32E-03 | IKZF3 |
| **rs4702698** |  | NS |  | 1.67E-03 | CMBL |
| **SRP54** | 2.26E-03 |  |  | 2.34E-03 |  |
| **rs7956536** |  | NS |  | 6.19E-03 | MVK |
| **ABTB1** | NS |  |  | 6.63E-03 |  |
| **rs917998** |  | NS |  | 8.21E-03 | IL18R1 |
| **rs5754467** |  | NS |  | NS |  |
| **HMGCR** | NS |  |  | NS |  |
| **rs2476601** |  | NS |  | NS |  |
| **rs2984920** |  | NS |  | NS |  |
| **rs4921293** |  | NS |  | NS |  |
| **rs9905921** |  | NS |  | NS |  |
| **NT5C1A** | NS |  |  | NS |  |
| **rs10189330** |  | NS |  | NS |  |
| **rs6599390** |  | NS |  | NS |  |
| **CHD4** | 1.16E-05 |  |  | ND |  |

Inputs were 19 myositis associated SNPs, 28 autoantibody targets and 19 SNPs and 27 autoantibody targets combined. (Due to the same candidate genes being selected from multiple inputs, CHD4, rs2286896, rs11724804 and rs3094013 were removed from the analysis). NS; not significant (p>0.01), ND; not determined.
