# Supplementary material for: Systematic protein-protein interaction and pathway analyses in the idiopathic inflammatory myopathies
Source: Arthritis Res Ther. 2016 Jul 7;18:156. doi: 10.1186/s13075-016-1061-7 (PMC4936183; doi:10.1186/s13075-016-1061-7)
Supplement: Additional file 5: Table S2. — Keywords strongly linking the significant genes in each region identified by text-based pathway analysis, using GRAIL. Inputs were 19 myositis-associated SNPs, 28 autoantibody targets and 19 SNPs and 27 auotantibody targets combined. Due to the same candidate genes being selected from multiple inputs, CHD4, rs2286896, rs11724804 and rs3094013 were removed from the analysis (DOCX 15.1 KB) [file 13075_2016_1061_MOESM5_ESM.docx]

**Table S2. Keywords strongly linking the significant genes in each region identified by text-based pathway analysis, using GRAIL.**

| **19 SNPs** | **28 MSA/MAAs** | **27 MSA/MAAs 19 SNPs** |
| --- | --- | --- |
| 'chemokine' | 'trna' | 'trna' |
| 'thymocytes' | 'synthetase' | 'synthetase' |
| 'autoimmune' | 'sumo' | 'sumo' |
| 'thymus' | 'yeast' | 'yeast' |
| 'diseases' | 'mismatch' | 'ubiquitin' |
| 'immune' | 'ubiquitin' | 'repair' |
| 'cells' | 'repair' | 'mismatch' |
| 'patients' | 'sumoylation' | 'enzyme' |
| 'responses' | 'cerevisiae' | 'cerevisiae' |
| 'mice' | 'chromatin' | 'sumoylation' |
| 'ligase' | 'intermediary' | 'anti' |
| 'production' | 'enzyme' | 'saccharomyces' |
| 'chemokines' | 'saccharomyces' | 'intermediary' |
| 'lymphoid' | 'nuclear' | 'degradation' |
| 'costimulatory' | 'complex' | 'conjugation' |
| 'arthritis' | 'proteins' | 'recognition' |
| 'activation' | 'conjugation' | 'scid' |
| 'tyrosine' | 'anti' | 'infection' |
| 'lupus' | 'complexes' | 'nuclear' |
| 'ubiquitin' | 'eukaryotic' | 'enzymes' |

Inputs were 19 myositis associated SNPs, 28 autoantibody targets and 19 SNPs and 27 auotantibody targets combined. (Due to the same candidate genes being selected from multiple inputs, CHD4, rs2286896, rs11724804 and rs3094013 were removed from the analysis). Keywords are listed in order of informativeness.
